# Editorial: Modulation of oxidative stress and inflammation via the NRF2 signaling pathway

**DOI:** 10.3389/fphar.2024.1483289

**Published:** 2024-09-17

**Authors:** Sarmistha Saha, Brigitta Buttari, Luciano Saso

**Affiliations:** ^1^ Department of Biotechnology, Institute of Applied Sciences and Humanities, GLA University, Mathura, Uttar Pradesh, India; ^2^ Department of Cardiovascular, Endocrine-Metabolic Diseases and Aging, Italian National Institute of Health, Rome, Italy; ^3^ Department of Physiology and Pharmacology “Vittorio Erspamer”, Sapienza University of Rome, Rome, Italy

**Keywords:** reactive oxygen species (ROS), inflammation, antioxidants, NRF2, oxidative stress

An imbalance between the formation of reactive oxygen species (ROS) and the reaction of antioxidant proteins is referred to as oxidative stress. A variety of biochemical, metabolic, and genetic pathways help cells to maintain this equilibrium of oxidants and antioxidants, and when it is out of balance, pathophysiological effects can result. Numerous pathogenic illnesses, including persistent infections (like HIV-1), inflammatory disorders, cardiovascular diseases, neurological diseases, and cancer, are thought to be triggered by ROS, reactive nitrogen (RNS), and reactive sulphur (RSS) species. Despite significant effort over the past few decades to translate antioxidant therapy into clinical practice, the majority of clinical trials utilizing general antioxidant therapy have failed, most likely as a result of a lack of understanding of the redox signaling pathways in health and disease. In fact, most antioxidant defense in cells is provided by antioxidant enzymes that use their unique substrates to decrease oxidants, not by exogenous or endogenous small molecules acting as scavengers. A logical strategy may be devised to enhance therapeutic intervention via a better understanding of the processes by which oxidants function as well as the limitations and potentials of antioxidant therapy.

Among the key antioxidant systems, Nrf2/Keap1 signaling systems have been shown to play a vital role. In order to maintain redox state and the production of antioxidant genes, Nrf2 and its antagonistic regulator, the E3 ligase adaptor Kelch-like ECH-associated protein 1 (Keap1), are essential. A transcription factor known as Nrf2 (NF-E2-related factor 2) is a member of the Cap'n'collar (CNC) family of bZIP transcription factors.

Under typical conditions, Keap1 binds to the Neh2 domain of Nrf2 via the ETGE and DLG motifs, causing Nrf2 to localize in the cytoplasm. However, the E3-ligase complex undergoes a conformational shift under oxidative stress, preventing Nrf2 from interacting with the ubiquitin-conjugating mechanism. As a result, the Nrf2 is liberated from the complex, moving into the nucleus where it forms a heterodimer with the sMaf protein before being activated by the ARE, which further controls the production of antioxidant proteins and cell defense systems. The Nrf2 pathway stimulates the expression of genes linked to NADH regeneration and redox detoxification, such as TXN, G6PD, GSTA2, NQO1, and HMOX1. Numerous oxidative stress-related diseases, such as neurodegenerative disorders, cardiovascular abnormalities, and pulmonary disorders, are linked to Nrf2 signaling. Nrf2 activators are therefore thought of as possible tools for increasing antioxidant capacity and treating disease. So far, the US Food and Drug Administration has approved dimethyl fumarate (DMF) and diroximel fumarate (DRF) as anti-inflammatory agents for multiple sclerosis treatment. These drugs have the capacity to reduce inflammation through the Nrf2 antioxidant pathway. A number of dietary Nrf2 activators, including curcumin, sulforaphane, and resveratrol, have been developed as daily supplements, while other Nrf2 activators are being tested in clinical settings as potential therapeutics. However, some challenges also exist. The majority of Nrf2 activators are electrophilic and quickly metabolized, which may result in limited bioavailability in distant organs and is connected to low effective biological concentration. Also, some Nrf2 activators may affect other signaling pathways and interfere with relevant biological processes in addition to activating Nrf2 and triggering antioxidant enzymes. This Research Topic shares insights on the state of the art for developing effective treatment approaches for the pharmacological control of inflammation and oxidative stress.

One of the key contributions to this Research Topic is the study by Chen et al., which describes the effect of a bicyclic terpenoid, Borneol, a traditional Chinese medicine, on the ROS generation in neutrophils. Neutrophil extracellular traps (NETs) can form in both infectious and non-infectious diseases and ROS production is related to the formation of NETs. Phenol-12-myristate-13-acetate was used to stimulate human neutrophils in order to cause NETosis; borneol pretreatment showed a protective mechanism. However, their study also revealed that the inhibition of NETosis by borneol is not dependent on Toll-like receptor 2/4.

Another compelling study by Cao et al. shows the protective effect of a triterpenoid compound, 18-α-glycyrrhetinic acid (GA), against periodontitis. In their research, GA successfully reduced H_2_O_2_-induced oxidative damage in human periodontal ligament cells by preventing Cx43 from being expressed and functioning. Cx43-mediated gap junctions play a crucial role in maintaining homeostasis. It was observed that GA suppressed the Cx43/JNK/NF-κB pathway and reduced apoptosis. However, there are still several challenges that exist which limit the use of GA as a specific drug. For this, there is a need for further detailed investigation of GA in genetically defective animal models.

Focusing on the Nrf2 signaling pathway, Bresciani et al. contribute a comprehensive review titled “*Novel potential pharmacological applications of dimethyl fumarate—an overview and update*.” DMF’s potential to treat a variety of ailments is drawing more and more attention. This review’s evidence supports the effectiveness of DMF-based treatments in many disease scenarios and makes a compelling case for the molecule’s increased scientific recognition. In fact, preclinical studies account for the majority of the summary findings, which serve as a foundation for more in-depth studies targeted at bolstering these findings and advancing them from the bench to the clinic.

Another review article by Soni et al. titled “*A critical appraisal of ferroptosis in Alzheimer’s and Parkinson’s disease: new insights into emerging mechanisms and therapeutic targets*.” provides a comprehensive evaluation of the unresolved molecular pathways that lead to the start and spread of ferroptosis, including the function of voltage-dependent anionic channels in mitochondrial membranes and a signaling cascade that de-represses lipoxygenase translation. Due to the dearth of effective disease-modifying medications, the majority of neurodegenerative disease treatments now in use focus on managing symptoms. Due to the important role ferroptosis plays in neurodegenerative diseases, Bach1/Nrf2-based therapeutics may provide a means of altering the disease by focusing on its underlying mechanisms. More efficient and potentially disease-modifying treatments that target ferroptosis for neurodegenerative illnesses will probably result from the discovery and optimization of this class of small molecules in the future.

The papers included in this Research Topic provide new directions for future advancements in therapeutic interventions as we continue to navigate the potentials and challenges in this Nrf2 signaling pathway. We anticipate that the articles in this Research Topic will stimulate greater investigation and cooperation, which will finally result in the development of more individualized and efficient treatment plans.

